# P-66. Comparison of Periprosthetic Tissue and Cement Spacer Sonicate Fluid Cultures at the Second Stage of Two-Stage Hip and Knee Revision Arthroplasty Surgery for Periprosthetic Joint Infection

**DOI:** 10.1093/ofid/ofae631.273

**Published:** 2025-01-29

**Authors:** Judith Alvarez Ortero, Derek Fleming, Melissa J Karau, Kerryl Greenwood-Quaintance, Matthew Abdel, Nicholas Bedard, Robin Patel

**Affiliations:** Mayo Clinic, Rochester, Minnesota; Mayo Clinic, Rochester, Minnesota; Mayo Clinic, Rochester, Minnesota; Mayo Clinic, Rochester, Minnesota; Mayo Clinic, Rochester, Minnesota; Mayo Clinic, Rochester, Minnesota; Mayo Clinic, Rochester, Minnesota

## Abstract

**Background:**

Chronic periprosthetic joint infection is commonly managed with two-stage exchange. Clinically, evaluation for persistent infection at the second stage of exchange may inform management. The aim of this study was to evaluate the yield of periprosthetic tissue and antimicrobial-containing cement spacer sonicate fluid cultures at the second stage of two-stage exchange surgeries.

**Methods:**

Periprosthetic tissue and sonicate fluid cultures of hip and knee cement spacers removed at the second stage of two-stage exchange surgeries between January 2010 and December 2020 were studied. Two or more positive tissue cultures yielding the same organism were considered positive. For sonicate culture, ≥20 CFU/10 ml of sonicate fluid was considered positive. Persistent infection was defined by the presence of at least one of the following: intraoperative purulence, sinus tract, histopathology showing acute inflammation, or clinical signs of infection. Cement spacers were removed as part of spacer exchange if persistent infection was suspected or for insertion of a new implant if infection was not suspected.

**Results:**

172 cultures were evaluated, 69% from knees and 31% from hips. Median patient age was 64 years; 53% were men. 38/172 (22%) of cement spacers were removed as part of a spacer exchange and 134/172 (78%) for insertion of a new implant. Persistent infection was present in 49/172 (28%). 18/172 (10%) received antibiotics 2 weeks before culture, including 8/18 (44%) undergoing spacer exchange. For persistent infections, tissue and sonicate culture were positive in 11/49 and 5/49, respectively. In the 123 without persistent infection, no tissue or sonicate cultures were positive. When a new implant was placed, persistent infection was observed in 18/134 (13%), and tissue and sonicate cultures were positive in 4/18 and 1/18, respectively. When a new implant was placed (116) and no infection was found, all tissue and sonicate cultures were negative. After spacer exchange, persistent infection was present in 31/38 (81%), and tissue and sonicate cultures were positive in 8/31 and 3/31, respectively; in the 7 without infection, there were no positive cultures.

**Conclusion:**

Sonication culture of cement spacers and periprosthetic tissue cultures have low sensitivities for persistent infection.

**Disclosures:**

**Matthew Abdel, MD**, Stryker: Advisor/Consultant|Stryker: Royalties **Nicholas Bedard, MD**, Stryker: Advisor/Consultant **Robin Patel, MD**, a patent on Bordetella pertussis/parapertussis PCR issued, a patent on a device/method for sonication with royalties paid by Samsung to Mayo Clinic, a: See above|MicuRx Pharmaceuticals and BIOFIRE: Grant/Research Support|PhAST, Day Zero Diagnostics, Abbott Laboratories, Sysmex, DEEPULL DIAGNOSTICS, S.L., Netflix, Oxford Nanopore Technologies and CARB-X: Advisor/Consultant|Up-to-Date and the Infectious Diseases Board Review Course.: Honoraria

